# Tetrandrine injection for preventing radiation-induced lung injury in patients with locally advanced esophageal squamous cell carcinoma: a randomized controlled trial protocol

**DOI:** 10.3389/fonc.2026.1895499

**Published:** 2026-07-28

**Authors:** Qianqian Liu, Xingping Tan, Wujie Wei, Fang Pan, Yifang Tang, Zijing Deng, Guanliang Wang

**Affiliations:** Department of Oncology, People’s Hospital of Chongqing Hechuan, Chongqing, China

**Keywords:** esophageal squamous cell carcinoma, radiation-induced lung injury, randomized controlled trial, study protocol, tetrandrine

## Abstract

**Background:**

Radiation-induced lung injury (RILI) is a common and dose-limiting toxicity in patients with locally advanced esophageal squamous cell carcinoma (ESCC) receiving concurrent chemoradiotherapy (CCRT). Effective prophylactic agents are lacking. Tetrandrine, a bisbenzylisoquinoline alkaloid, has shown anti-inflammatory and anti-fibrotic properties in preclinical studies. This randomized controlled trial aims to evaluate the efficacy and safety of tetrandrine injection for preventing RILI in this population.

**Methods:**

This is a single-center, randomized, placebo-controlled, double-blind, phase II trial. A total of 100 eligible patients with locally advanced ESCC (T3-4N+M0) receiving definitive CCRT will be randomly assigned (1:1) to receive either intravenous tetrandrine (200–300 mg/day, 6 days per week for 12 weeks) or matching placebo. Radiotherapy dose is 50–50.4 Gy (1.8 Gy/fraction) using IMRT, with concurrent docetaxel/cisplatin. The primary endpoint is the incidence of grade ≥2 RILI according to CTCAE v5.0. Secondary endpoints include any-grade RILI, time to RILI onset, change in pulmonary function, and CCRT completion rate. Sample size calculation (40 evaluable per group) provides 80% power to detect a 15% absolute risk reduction (from 25% to 10%) with two-sided α=0.20. The protocol follows SPIRIT 2013 guidelines. Recruitment has not yet started.

**Discussion:**

This trial will generate preliminary evidence on the efficacy of tetrandrine for preventing RILI in ESCC patients. By adopting internationally accepted radiotherapy doses (50–50.4 Gy) and stringent lung dose constraints (V20 ≤ 30%, MLD < 20 Gy), the study provides a robust framework for comparing outcomes with global literature.

**Systematic review registration:**

https://www.chictr.org.cn/bin/project/edit?pid=289682, identifier ChiCTR2500111007.

## Introduction

Esophageal cancer ranks among the top ten most common malignancies globally. According to GLOBOCAN 2023 data, there are over 470,000 new cases and 370,000 deaths annually worldwide. More than 90% of these are esophageal squamous cell carcinomas (ESCC) (1). For patients with locally advanced ESCC (clinical stage T3-4N0-1M0), where the tumor exhibits deep invasion or regional lymph node involvement, surgical resection alone is often insufficient for curative treatment. Consequently, concurrent chemoradiotherapy has become the standard of care for this patient group (2).

Radiotherapy works by delivering precisely targeted radiation to eradicate tumor cells, significantly improving local control rates. It raises the two-year survival rate from approximately 35% with surgery alone to over 50%. However, the anatomical proximity of the esophagus to the lungs means irradiation inevitably exposes surrounding healthy lung tissue, leading to radiation-induced lung injury (RILI). This is a major clinical challenge that limits the radiotherapeutic efficacy and adversely affects patients’ quality of life. RILI represents a critical dose-limiting toxicity, with reported incidence rates ranging from 10.7% to 35.1% (3, 4). More critically, the occurrence of RILI during radiotherapy can cause treatment delays or even discontinuation. This not only compromises local tumor control but also increases the economic burden on patients and, in severe cases, can be life-threatening.

Radiation-induced lung injury (RILI) typically evolves through two distinct phases: an acute radiation pneumonitis phase and a subsequent chronic pulmonary fibrosis phase. The acute phase commonly occurs 1 to 3 months after radiotherapy, presenting clinically with symptoms such as cough, dyspnea, and fever. Imaging studies typically reveal ground-glass opacities or consolidations in the irradiated lung fields. If the condition progresses, it transitions into the chronic fibrotic phase 6 to 12 months post-radiation, characterized by irreversible fibrotic tissue proliferation and a progressive decline in pulmonary function. In severe cases, this may advance to respiratory failure and even death. Clinical data indicate that the incidence of RILI following radiotherapy in patients with locally advanced esophageal squamous cell carcinoma (ESCC) is as high as 30% to 50%, with moderate to severe (grade ≥2) injury occurring in approximately 15% to 20% of patients (5). Moderate-to-severe RILI not only leads to persistent symptoms such as dyspnea and chest pain, significantly impairing quality of life (as evidenced by a 20- to 30-point reduction in EORTC QLQ-C30 scores compared to those without injury), but may also necessitate interruption or reduction of radiotherapy dosage. Studies have shown that treatment interruption due to RILI is associated with an 18% to 25% increase in local tumor recurrence rates and a 10% to 15% decrease in 5-year survival rates (6). Consequently, identifying effective strategies to prevent or mitigate radiotherapy-related RILI in patients with locally advanced ESCC has emerged as a critical clinical challenge in the field of radiation oncology.

Currently, the clinical strategies available for preventing radiation-induced lung injury (RILI) remain limited. Although corticosteroids are widely used in the treatment of acute radiation pneumonitis, they are not recommended as routine prophylactic agents due to their potential to suppress immune function, increase infection risk, and inability to delay the progression of chronic fibrosis, as advised by the Chinese Guidelines for Radiotherapy of Esophageal Cancer (2023). Amifostine, a broad-spectrum radioprotector, has demonstrated some lung protection in animal studies; however, clinical research indicates that its preventive effect against chronic/late pulmonary toxicity is not significant (RR = 0.74, 95% CI: 0.45–1.19; P = 0.210) (7). Its clinical application is further limited by gastrointestinal adverse effects such as nausea and vomiting (8, 9). Additionally, the preventive efficacy of other agents—including antioxidants (e.g., vitamin E, N-acetylcysteine) and cytokine inhibitors (e.g., transforming growth factor-beta antagonists)—remains under investigation in clinical trials, and no consensus has been reached to support their routine clinical use. Therefore, there is a pressing clinical need and significant research value in identifying safe and effective agents for the prevention of RILI, particularly those aligned with the clinical context in China and suitable for patients with esophageal squamous cell carcinoma (ESCC).

Tetrandrine (TET), a bisbenzylisoquinoline alkaloid extracted from the root of the traditional Chinese medicine Stephania tetrandra S. Moore, exhibits multiple pharmacological activities including anti-inflammatory, antioxidant, and anti-fibrotic effects. It has attracted broad attention for its potential applications in the field of lung diseases. Recent basic research has demonstrated that tetrandrine can exert lung-protective effects through multiple mechanisms: on one hand, it inhibits radiation-induced apoptosis of pulmonary epithelial cells and downregulates the expression of pro-inflammatory cytokines such as tumor necrosis factor-α (TNF-α) and interleukin-6 (IL-6), thereby alleviating pulmonary inflammation (10); on the other hand, tetrandrine suppresses the proliferation and activation of lung fibroblasts and reduces collagen synthesis and deposition, thus delaying or preventing the progression of pulmonary fibrosis (11). In clinical practice, tetrandrine tablets have been used for the treatment of silicosis and pulmonary fibrosis, demonstrating favorable safety and tolerability. Meanwhile, tetrandrine injection, as an intravenous formulation, offers advantages such as high bioavailability and stable blood concentration, making it more suitable for preventing radiation-induced lung injury during radiotherapy where rapid onset of action is required.

Our preliminary small-scale clinical observation indicated that among patients receiving radiotherapy for thoracic tumors (such as lung cancer and esophageal cancer), the use of tetrandrine injection reduced the incidence of RILI from 45% to 28%. However, as the study was a retrospective analysis with a small sample size (n = 32) and lacked a strictly designed control group, the reliability and generalizability of the findings are limited.

Therefore, this study aims to adopt a randomized controlled trial design to investigate the preventive effect of tetrandrine injection on RILI in patients with locally advanced ESCC undergoing radiotherapy. We hypothesize that, compared with conventional care alone, the combined use of tetrandrine injection during radiotherapy will significantly reduce the incidence of grade ≥2 RILI, improve pulmonary function, and enhance quality of life in this patient population.

The implementation of this study is expected to provide a new and effective pharmacologic option for preventing RILI, further optimizing the comprehensive treatment strategy for locally advanced ESCC, and ultimately improving survival outcomes and quality of life for patients. To bridge this evidence gap, we designed this prospective, double-blind, phase II pilot/feasibility RCT to evaluate whether tetrandrine injection can reduce moderate-to-severe RILI and to generate preliminary data on safety, adherence, and recruitment to inform a future definitive trial.

## Methods

### Study design and participants

**Figure 1:**
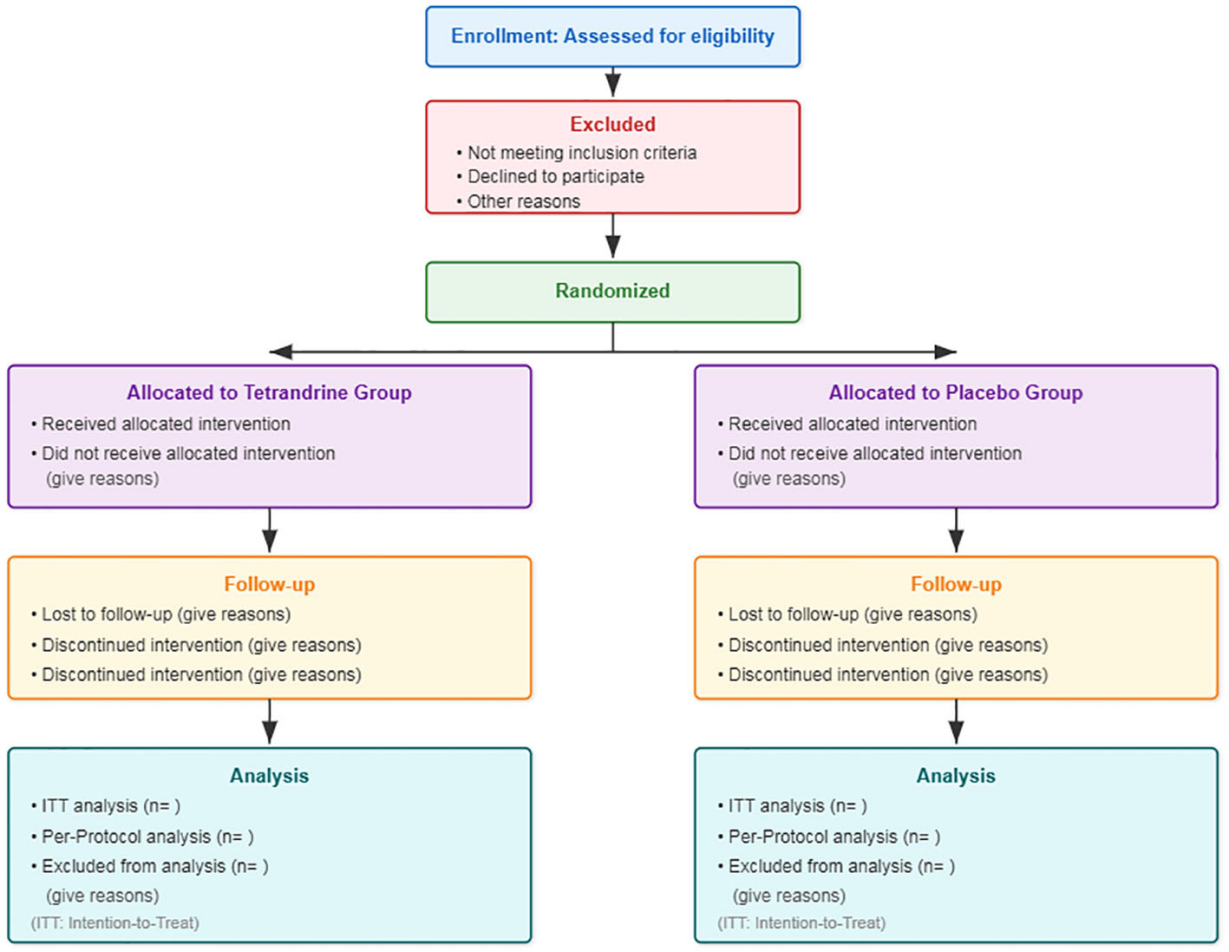
CONSORT flow diagram of the randomized controlled trial. This figure shows a pre-specified CONSORT flow diagram template. Actual patient numbers will be reported in the final study report. ITT, intention-to-treat.

This is a randomized, placebo-controlled, double-blind, single-center, phase II clinical trial designed to evaluate the potential of tetrandrine in preventing radiation-induced lung injury (RILI) in patients with locally advanced esophageal squamous cell carcinoma (ESCC) undergoing definitive concurrent chemoradiotherapy (CCRT). Participants will be randomly assigned in a 1:1 ratio to either the tetrandrine group or the control group. A schematic diagram of the study flow is presented in **Figure 1**.

Detailed inclusion, exclusion, and withdrawal criteria are presented in **Table 1**.

**Table 1:** Inclusion,exclusion and withdrawal criteria.

Inclusion criteria	Exclusion criteria	Withdrawal criteria
Age between 18 and 75 years old.	History of chest radiotherapy.	Treatment delay for more than 2 weeks or interruption due to extended toxicity.
ECOG performance status of 0–1.	Concomitant tumor in other organs or prior malignancies within 5 years.	Disease progression as judged by researchers during the treatment.
Pathological confirmation of ESCC.	History of interstitial lung disease or non-infectious pneumonia including chronic obstructive pulmonary disease.	
Imaging staging suggesting T3-4N+M0 thoracic esophageal cancer according to AJCC eight staging criteria (primary lesions mainly in the chest cavity).	Severe cardiovascular disease, cerebrovascular complications, epilepsy, active peptic ulcers, infections, psychological disorders or other serious underlying diseases that may limit the patient’s understanding and tolerance of comprehensive treatments.	
No restriction on the number of regional nodal stations or bulk of disease.	A previous history of ataxia due to telangiectasia or other radiosensitivity reaction.	
No ability to tolerate surgery or tumor not surgically removable.	Scleroderma or active connective tissue disease.	
No perforation or deep niche in the esophageal cancer lesion.		
No prior chemotherapy, immunotherapy or surgery before radiotherapy.		
Ability to eat liquid foods.		
Adequate bone marrow, liver, kidney, blood coagulation and lung functions.		
Life expectancy of >6 months.		
Voluntary participation in the study, with good compliance.		

All eligible patients must provide written informed consent and commit to participate in and complete the study, including follow-up.

#### Pre-treatment

##### Assessment and screening

Each patient must undergo the following examinations and consultations within 1 or 2 weeks prior to trial initiation:

1. Detailed medical history review.
2. Physical examination to document height, weight, vital signs, and ECOG performance status score.
3. Blood tests including complete blood count (CBC), serum biochemistry, coagulation profile, hepatitis B virus (HBV) screening and HBV DNA (for HBsAg-positive patients), as well as esophageal cancer-related tumor markers such as carcinoembryonic antigen, cancer antigen (CA) 19-9, CA 125, CA 15-3, neuron-specific enolase, squamous cell carcinoma antigen, soluble cytokeratin 19 fragment, and alpha-fetoprotein. While not all markers are specific to ESCC, this comprehensive panel is collected as part of routine institutional practice for baseline health assessment and to facilitate exploratory analyses of potential prognostic factors for tumor response and toxicity in our cohort.
4. Cardiopulmonary function assessment, including electrocardiogram, echocardiogram, and pulmonary function tests.
5. Upper gastrointestinal endoscopy with biopsy.
6. Imaging studies including contrast-enhanced chest/abdominal CT, upper gastrointestinal series with meglumine diatrizoate and simethicone, pelvic CT with contrast if clinically indicated, and [¹ ^5^ F]fluorodeoxyglucose positron emission tomography/CT (FDG-PET/CT) for more accurate staging if consented by the patient.
7. Nutritional assessment and counselling.
8. Smoking cessation counselling.

#### Radiotherapy

##### Target volume delineation principles

The gross tumor volume (GTV) includes the primary tumor (GTVp) and enlarged regional lymph nodes (GTVn), as determined by CT simulation scanning and other diagnostic imaging modalities. Elective nodal irradiation (ENI) will be applied for clinical target volume (CTV) delineation.

The CTV for GTVp is defined as the primary tumor volume with 3 cm superior and inferior margins, plus a 0.5–1 cm radial expansion encompassing peri-esophageal lymph nodes. The CTV for GTVn should cover all involved lymph nodes and high-risk lymphatic drainage areas, such as supraclavicular regions for upper and mid-thoracic tumors.

The planning target volume (PTV) for both GTV and CTV is generated by applying a uniform 0.5 cm margin in all directions. The prescribed dose is 50.4 Gy in 28 fractions (1.8 Gy per fraction) to all PTVs. If target volume coverage is compromised due to proximity to organs at risk, the radiation oncologist will decide whether to irradiate only PTV-GTV or PTV-CTV. At least 95% of each PTV should receive the prescribed dose. Dose constraints for normal tissues are detailed in **Table 2**.

**Table 2:** Standardized dose constraints for organs at risk (OAR).

OAR	Constraint	Priority	Clinical rationale
Lung	V5 ≤ 63%	Mandatory	Minimize low-dose irradiation to large lung volumes
	V20 ≤ 30%	Mandatory	Keep risk of grade ≥2 radiation pneumonitis < 20%
	MLD < 20 Gy	Mandatory	Standard predictor of pneumonitis risk
Heart	V30 < 46%	Mandatory	Reduce cardiac morbidity and mortality
	V25 < 10%	Mandatory	Constrain moderate-dose exposure
	Mean < 26 Gy	Mandatory	Overall heart dose limit
Spinal cord	Cervical: Max ≤ 45 Gy	Mandatory	Prevent myelopathy
	Thoracic: Max ≤ 50 Gy	Mandatory	Prevent myelopathy
Stomach	V40 < 40%–50%	Recommended	Reduce gastritis and ulcer risk
	Max ≤ 55–60 Gy	Recommended	Limit maximum point dose
Liver	Mean ≤ 30–32 Gy	Recommended	Preserve hepatic function

All constraints listed as “Mandatory” are non-negotiable and must be met in all treatment plans. Any protocol deviation from these mandatory constraints must be pre-approved by the principal investigator, with the reason for deviation and risk-benefit assessment documented in the patient’s radiotherapy file. “Recommended” constraints should be met whenever clinically feasible without compromising target coverage. Deviations from recommended constraints will be recorded and included in the dosimetric data analysis.

All patients will be positioned supine on the treatment couch and immobilized using customized thermoplastic masks. Image-guided radiotherapy will be performed daily with cone-beam CT (CBCT) for positional verification. Intensity-modulated radiation therapy (IMRT) is recommended for treatment planning.

Dose calculation in regions with artefacts may lead to inaccuracies in dose distribution and high-dose deviations. To ensure radiotherapy quality, the Acuros XB algorithm (version 15) will be used for precise dose computation. This algorithm has been reported to be as accurate as or comparable to Monte Carlo simulation. All treatment plans will be verified using a Delta4 Phantom, with a gamma passing rate >98% under the 3%/3 mm criterion.

### Dosimetric data collection

To enable comprehensive analysis of factors associated with RILI risk, the following dosimetric variables will be systematically extracted from the treatment planning system for each patient:

1. Lung dosimetric parameters: V5, V10, V20, V30 (percentage of lung volume receiving ≥5 Gy, ≥10 Gy, ≥20 Gy, and ≥30 Gy, respectively), and mean lung dose (MLD).
2. Target volume parameters: Planning target volume (PTV) for GTV (PTV-GTV) and PTV for CTV (PTV-CTV), expressed in cubic centimetres (cm³).
3. Anatomical parameters: Total lung volume (cm³) and tumor location classified as upper thoracic (above the carina), middle thoracic (at the level of the carina), or lower thoracic (below the carina).
4. Other relevant parameters: Maximum dose to the spinal cord and mean heart dose.

These dosimetric and anatomical variables will be collected prospectively and included as covariates in multivariable regression models to adjust for potential confounding and to explore their associations with RILI outcomes. The correlation between dose-volume parameters and RILI grade will be analyzed using Spearman’s rank correlation or multivariate logistic regression, as appropriate.

### Chemotherapy

Patients will receive two cycles of chemotherapy consisting of intravenous docetaxel (60 mg/m² on day 1) and cisplatin (30 mg/m² on days 1 and 2).Pegylated recombinant human granulocyte colony-stimulating factor (Pegfilgrastim, 6 mg, subcutaneous, once per cycle, 24-72 hours post-chemotherapy) is strongly recommended for primary prophylaxis against neutropenia to minimize treatment delays during concurrent chemoradiation.

Dose adjustments will be based on hematological and biochemical parameters from the previous cycle. Subsequent chemotherapy cycles will be administered provided the following criteria are met: (1) absolute neutrophil count ≥1.5 × 10^9^/L; (2) platelet count ≥100 × 10^9^/L; (3) alanine aminotransferase, aspartate aminotransferase, and total bilirubin levels within Common Terminology Criteria for Adverse Events (CTCAE)-defined limits; and (4) recovery of non-hematological toxicities (except alopecia) to grade 1 or baseline.

In cases of grade 3 or higher toxicities, doses of both agents will be reduced by 20%. Radiotherapy and concurrent chemotherapy will be discontinued in the event of grade 3 or 4 radiation pneumonitis.

#### Randomization and intervention

Participants will be randomly assigned in a 1:1 ratio to either the tetrandrine or control group using a computer-generated randomization sequence. All participants and treating physicians will be blinded to the treatment allocation.

Jiangxi Yintao Pharmaceutical Co., Ltd. will supply identical vials of tetrandrine injection and matching placebo. Both preparations are indistinguishable in appearance and pharmaceutical properties.

Placebo composition and packaging: The placebo will be composed of the same vehicle and excipients as the tetrandrine injection (5% glucose or normal saline with stabilisers) but will not contain the active pharmaceutical ingredient. Both tetrandrine and placebo will be supplied in identical glass vials that are visually indistinguishable in terms of color, transparency, labelling, and packaging. The vials will be packaged in sequentially numbered, opaque containers according to the computer-generated randomization list, ensuring concealment of allocation.

Administration and blinding maintenance: Both tetrandrine and placebo will be diluted in 250 mL of 5% glucose or normal saline and administered via identical infusion sets over the same duration (1–2 hours) by slow intravenous infusion. The infusion rate will be standardized across both groups to minimize the risk of unblinding due to infusion-site symptoms. Patients in both arms will be monitored equally for infusion-related adverse events. To maintain blinding, the investigator responsible for assessing RILI outcomes (including the multidisciplinary adjudication committee and central radiology reviewers) will be separated from the clinical team administering the treatment. The allocation code will be kept strictly confidential and will only be broken in case of a medical emergency where knowledge of the treatment assignment is essential for patient safety.

Tetrandrine will be administered at a daily dose of 200–300 mg, diluted in 5% glucose or normal saline, and delivered by slow intravenous injection or infusion. The drug will be given for 6 consecutive days followed by a 1-day break per week. Tetrandrine treatment will commence on the first day of radiotherapy and continue for 12 weeks.

Rationale for dose and duration: The dose range of 200–300 mg/day is selected based on the standard clinical dose of tetrandrine injection used for silicosis and other fibrotic conditions in China, as well as the dose used in our preliminary observational study. This dose has demonstrated favourable safety and tolerability in clinical practice. The 12-week treatment duration was chosen to cover the entire course of concurrent chemoradiotherapy (approximately 5–6 weeks) and the subsequent 6-week period immediately following radiotherapy, which represents the peak window for the onset of radiation pneumonitis.

Known toxicity profile: At the therapeutic dose range (200–300 mg/day), adverse reactions have rarely been reported in clinical use. However, higher doses have been associated with toxicity: doses of 528 mg have been associated with hemoglobinuria and mild anemia; 675 mg may cause dizziness, nausea, vomiting, chills, respiratory distress, and suffocation; and 840 mg has been linked to acute glomerular necrosis. Pain or phlebitis may occur at the injection site. Tetrandrine is contraindicated in patients with known hypersensitivity to the drug.

#### Concomitant medication

Concomitant use of agents with potential antifibrotic activity—including amifostine and thalidomide—is prohibited. Medications that may increase the risk of tetrandrine-related adverse effects or reduce its efficacy should also be avoided.

#### Endpoints

RILI is primarily an imaging diagnosis established after clinical suspicion and exclusion of alternative pulmonary pathologies (e.g., pre-existing lung disease or infection). It often presents without specific symptoms, vital sign alterations, laboratory abnormalities, or distinctive imaging findings. Awareness of the radiation history and possible RILI is essential during routine follow-up.

Blinded independent adjudication: Diagnosis and grading must be confirmed through multidisciplinary consultation involving radiation oncologists, pulmonologists, and radiologists. To ensure unbiased assessment, all members of the multidisciplinary adjudication committee will be blinded to treatment allocation. Treatment assignment will not be disclosed to the committee members during the diagnostic process. At each follow-up, whenever feasible, patients will be evaluated via face-to-face assessment including medical history, physical signs, chest CT scans, and review of prior radiotherapy fields. RILI will be scored according to CTCAE v5.0, with imaging features used to classify the injury into one of five grades.

Central radiology review: A central, independent radiology review will be implemented to ensure consistent and objective imaging assessment. All chest CT scans—both baseline scans and those performed at the time of suspected RILI—will be reviewed by at least two independent radiologists who are blinded to treatment allocation and clinical details. The reviewing radiologists will not be involved in patient care and will have no access to treatment assignment information. Discordant readings between the two reviewers will be resolved by consensus; if consensus cannot be reached, a third senior radiologist will be consulted as the final adjudicator. The central review process will follow a standardized imaging assessment form that documents predefined radiological features of RILI (ground-glass opacities, consolidations, fibrotic changes, and their distribution relative to the radiation field).

Differential diagnosis procedures: To ensure the accuracy of RILI diagnosis, a systematic approach will be adopted to exclude alternative pulmonary conditions:

1. Exclusion of infection: All patients presenting with suspected RILI will undergo comprehensive clinical evaluation, including assessment of vital signs (temperature, heart rate, respiratory rate), laboratory tests (complete blood count, C-reactive protein, procalcitonin), and sputum culture and Gram staining. Patients with fever (≥38.5 °C), elevated procalcitonin (>0.5 ng/mL), or positive sputum cultures will be considered to have possible pulmonary infection and will be treated with antibiotics accordingly. These patients will be re-evaluated after 7–10 days of antibiotic therapy; only those without clinical and radiological improvement following adequate antibiotic treatment will be considered for RILI diagnosis.
2. Exclusion of tumor progression: Chest CT scans at the time of suspected RILI will be compared with baseline and mid-treatment imaging to assess whether new opacities appear within or outside the radiation field. Lesions located outside the radiation field, progressive mass-like consolidations, or new extrapulmonary metastases will suggest tumor progression rather than RILI. If tumor progression is suspected, further evaluation with FDG-PET/CT or biopsy will be performed as clinically indicated.
3. Exclusion of aspiration pneumonia: Patients with a history of dysphagia, esophageal stricture, or evidence of aspiration on imaging (dependent opacities in the posterior lung segments) will be evaluated for aspiration pneumonia. A detailed history of feeding status, swallowing difficulties, and episodes of choking during meals will be obtained. If aspiration is suspected, swallowing assessment and dietary modification will be recommended, and the patient will be monitored for response to aspiration precautions.
4. Exclusion of chemotherapy-related pneumonitis: Although the chemotherapy regimen (docetaxel/cisplatin) is not classically associated with high rates of pneumonitis, the possibility of drug-induced interstitial lung disease will be considered. Features suggestive of chemotherapy-related pneumonitis include diffuse bilateral opacities without clear radiation field correlation, peripheral distribution, and temporal association with chemotherapy administration. The oncology team will be consulted to evaluate this possibility, and chemotherapy may be held if drug-induced lung injury is suspected.
5. Exclusion of postoperative pulmonary complications: As this trial exclusively enrolls patients receiving definitive CCRT, surgical resection is not performed within the study protocol. Therefore, postoperative pulmonary complications are not applicable to this patient population.

Secondary endpoints also include change in quality of life (QoL) as assessed by the European Organisation for Research and Treatment of Cancer (EORTC) Quality of Life Questionnaire Core 30 (QLQ-C30) and the EORTC QLQ-OES18 (esophageal cancer-specific module). These questionnaires will be administered at baseline, at the end of radiotherapy (12 weeks), and at 6-month and 12-month follow-up visits. Changes in QoL scores from baseline to each time point will be compared between the tetrandrine and placebo groups.

**Figure 2:**
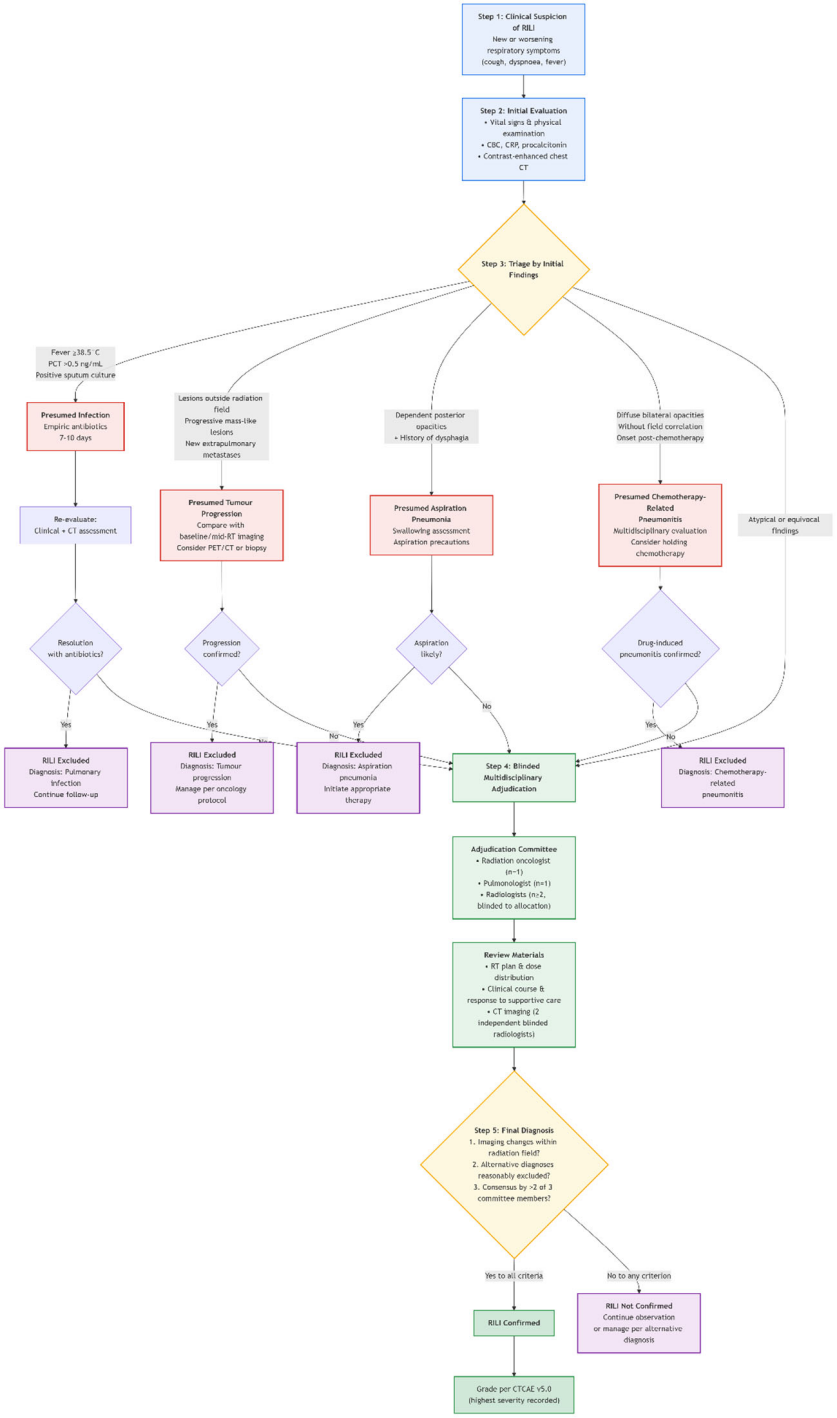
Diagnostic algorithm for radiation-induced lung injury (RILI) adjudication. The algorithm outlines a stepwise, standardized approach for evaluating patients with clinically suspected RILI.

Diagnostic algorithm: A diagnostic algorithm will guide the adjudication process (**Figure 2**). Patients with suspected RILI will first undergo evaluation for infection and aspiration. If infection is excluded, the case will be reviewed by the blinded multidisciplinary committee. RILI will be confirmed only when alternative diagnoses have been reasonably excluded and imaging changes are consistent with radiation-induced injury within the irradiated lung volume.

Step 1: clinical suspicion based on new or worsening respiratory symptoms.

Step 2: initial evaluation including physical examination, laboratory tests (complete blood count, C-reactive protein, procalcitonin), and contrast-enhanced chest computed tomography (CT).

Step 3: systematic triage to exclude alternative diagnoses—pulmonary infection (empiric antibiotics with re-evaluation), tumor progression (comparison with baseline imaging, PET/CT or biopsy if indicated), aspiration pneumonia (swallowing assessment), and chemotherapy-related pneumonitis (multidisciplinary evaluation).

Step 4: blinded multidisciplinary adjudication by a committee comprising a radiation oncologist, a pulmonologist, and at least two independent radiologists who are masked to treatment allocation.

Step 5: final diagnosis confirmed only when imaging changes are localized within the radiation field, alternative diagnoses have been reasonably excluded, and consensus is reached by at least two of the three committee members. RILI is graded according to Common Terminology Criteria for Adverse Events (CTCAE) version 5.0. CRP, C-reactive protein; CT, computed tomography; Dx, diagnosis; PCT, procalcitonin; PET/CT, positron emission tomography/computed tomography; RT, radiotherapy.

Dyspnea and dry cough are the most frequent symptoms of acute lung injury. Occasional fever is generally mild, though high fever may suggest concurrent infectious pneumonia. Chronic radiation fibrosis (RF) is a slowly progressive respiratory condition that may lead to respiratory insufficiency. Physical examination may be unremarkable or reveal pleural rubs, crackles, or signs of consolidation.

Chest imaging—particularly lung CT at baseline and during follow-up—is crucial for diagnosis and grading.

In the acute phase (typically 4–8 weeks post-radiotherapy), CT may show exudative changes within the radiation field, appearing as patchy or fluffy ground-glass opacities with ill-defined margins.

In the consolidation phase (usually 2–3 months post-radiotherapy), CT may demonstrate irregular high-density consolidations that do not conform to lobar or segmental anatomy, sometimes with air bronchograms.

In the fibrotic phase (generally ≥6 months post-radiotherapy), CT often reveals reticulation, cord-like or patchy dense shadows with sharp margins, accompanied by pleural thickening, reduced lung volume, hilar retraction, ipsilateral vascular narrowing, and compensatory contralateral hyperinflation.

Given tetrandrine’s anti-inflammatory and antifibrotic properties, cases exhibiting both radiation pneumonitis (RP) and RF will be graded according to the highest severity observed. For example, if a patient has grade 3 RP and grade 2 RF, the primary endpoint will be recorded as grade 3. Since tetrandrine is a promising agent for preventing both RP and RF, a 1-year observation period was selected to capture the occurrence of both acute and late radiation-induced lung injuries.

##### Safety monitoring and stopping rules

Data Safety Monitoring Board (DSMB): A independent Data Safety Monitoring Board will be established to oversee the safety of this trial. The DSMB will comprise three members: a radiation oncologist, a clinical pharmacologist, and a biostatistician, none of whom have conflicts of interest with the study. The DSMB will review accumulating safety data at predefined intervals: (1) after the first 10 participants have completed 4 weeks of treatment, (2) after 50% of participants have completed the intervention period, and (3) upon any serious adverse event (SAE) report. The DSMB will have the authority to recommend modification, suspension, or termination of the trial based on safety findings.

Stopping rules for the trial: The trial will be stopped prematurely if any of the following criteria are met:

1. The incidence of grade 4 or 5 adverse events (CTCAE v5.0) judged as related to tetrandrine exceeds 10% in the tetrandrine group.
2. Two or more deaths judged as related to tetrandrine occur within the first 6 months of the trial.
3. An independent DSMB recommendation for termination based on a pre-specified interim safety analysis.

Criteria for dose interruption and discontinuation for individual participants:

1. Dose interruption: Treatment with tetrandrine will be temporarily interrupted if a participant experiences any grade 3 tetrandrine-related adverse event (except grade 3 radiation pneumonitis, which will be managed per radiotherapy protocol). Interruption will continue until the adverse event recovers to grade ≤1 or baseline. After recovery, tetrandrine may be resumed at the same dose at the discretion of the investigator.
2. Permanent discontinuation: Tetrandrine will be permanently discontinued if a participant experiences: (a) any grade 4 tetrandrine-related adverse event; (b) a second episode of grade 3 tetrandrine-related adverse event after dose interruption and rechallenge; (c) grade 3 or 4 hypersensitivity reaction; (d) any tetrandrine-related adverse event that, in the investigator’s judgment, poses an unacceptable risk to the participant’s safety.

Adverse event reporting: All adverse events will be recorded from the time of randomization until 30 days after the last dose of tetrandrine. Serious adverse events (SAEs) will be reported to the ethics committee within 24 hours of the investigator becoming aware of the event, and to the regulatory authority within the timeframe required by local regulations. All AEs and SAEs will be followed until resolution or stabilization, with the outcome documented. The DSMB will be notified of all SAEs and will review the cumulative safety data regularly. Annual safety reports will be submitted to the ethics committee in accordance with institutional requirements.

### Follow-up and assessments

During the intervention period

1. Complete blood count (CBC) and biochemical analyses will be performed weekly.
2. Physical examination and nutritional assessment will be conducted on a weekly basis.
3. Contrast-enhanced chest/abdominal CT scans will be performed after 23 fractions of radiotherapy to evaluate tumor response and assess eligibility for surgery.

Pulmonary function testing (PFT): PFT will be performed at three prespecified timepoints: (1) baseline (within 1–2 weeks prior to the start of radiotherapy), (2) 3 months post-radiotherapy, and (3) 12 months post-radiotherapy. The following parameters will be measured: forced vital capacity (FVC), forced expiratory volume in 1 second (FEV1), FEV1/FVC ratio, and carbon monoxide diffusing capacity (DLCO). Changes in PFT parameters from baseline will be compared between the two groups.

Quality of life assessment: QoL will be assessed using the EORTC QLQ-C30 (version 3.0) and the EORTC QLQ-OES18. These validated instruments will be administered at four timepoints: (1) baseline, (2) at the end of radiotherapy (week 12), (3) 6 months post-radiotherapy, and (4) 12 months post-radiotherapy. All questionnaires will be self-administered by patients in a quiet environment, with assistance available from trained research staff if needed. The QLQ-C30 includes five functional scales (physical, role, cognitive, emotional, and social), three symptom scales (fatigue, nausea/vomiting, pain), a global health status/QoL scale, and six single items (dyspnoea, insomnia, appetite loss, constipation, diarrhoea, financial difficulties). The QLQ-OES18 assesses esophageal cancer-specific symptoms including dysphagia, eating difficulties, reflux, and pain. Scoring will follow the EORTC scoring manual, with all scales linearly transformed to a 0–100 range.

### Post-treatment follow-up

Patients will be followed for 1 year or until death. The first follow-up visit will occur 1 month after radiotherapy, followed by assessments every 3 months thereafter. The interval may be extended based on clinical indications. Routine evaluations include assessment of clinical symptoms, blood tests (including tumor markers), contrast-enhanced CT, upper gastrointestinal contrast studies, and pulmonary function tests. Endoscopic surveillance is recommended annually within the first postoperative year. If recurrence is suspected, physical examination, imaging studies, and pathological biopsy will be performed.

### Statistical analysis

Based on retrospective data from the authors’ institution and previous literature, the incidence of grade ≥2 radiation-induced lung injury is assumed to be 25% in the control group and 10% in the tetrandrine group. This randomized phase II trial is designed with a two-sided α of 0.20 and 80% power. Sample size calculation was performed using PASS software (v15.0.5). According to the phase II screening design, subsequent phase III studies would require a stricter α level (e.g., < 0.05). It should be noted that positive results from phase II trials—even if promising—are generally not considered definitive without phase III validation.

A total of 50 participants per group will be enrolled. Accounting for an estimated dropout rate of 20%, approximately 40 participants per group are expected to be evaluable for the primary endpoint.

All analyses will be conducted using SAS version 9.3 (SAS Institute, Cary, NC, USA). Continuous variables will be compared using t-tests, and categorical variables will be analyzed with chi-square or Fisher’s exact tests. Survival rates will be estimated using the Kaplan–Meier method and compared with the log-rank test. Confidence intervals for survival distributions will be calculated using the Greenwood formula. Hazard ratios (HR) will be derived from Cox proportional hazards models. A p-value < 0.05 will be considered statistically significant. Given the pilot nature of this trial, all secondary endpoints will be reported with point estimates and 95% confidence intervals, without adjustment for multiple comparisons.

Stratification and adjusted analyses: Participants will be stratified at randomization by baseline pulmonary function (FEV1/FVC ratio ≥70% versus <70%) to ensure balance between the two groups. To account for potential confounding, multivariable logistic regression models will be used to estimate the independent effect of tetrandrine on the primary endpoint (incidence of grade ≥2 RILI). The following prespecified covariates will be included in the adjusted model:

1. Baseline pulmonary function (FEV1/FVC ratio, continuous variable).
2. Smoking status (never smoker, former smoker, current smoker).
3. Tumor location (upper thoracic, middle thoracic, lower thoracic).
4. Mean lung dose (MLD, continuous variable).
5. V20 (continuous variable).
6. Age (continuous variable).

Each covariate was selected based on its established association with RILI risk in the literature. Variables with missing data will be handled using multiple imputation or complete-case analysis as sensitivity analyses. The multivariable models will be checked for multicollinearity using variance inflation factors (VIF), with VIF > 10 indicating potential collinearity.

Subgroup analyses: Exploratory subgroup analyses will be performed to assess the consistency of treatment effects across key RILI risk factors, including:

1. Smoking status (never smokers versus ever smokers).
2. Tumor location (upper/mid-thoracic versus lower thoracic).
3. Presence of baseline COPD (yes versus no).

These subgroup analyses are exploratory in nature and will be interpreted cautiously. Interaction tests will be performed to evaluate whether treatment effects differ significantly across subgroups. All subgroup results will be presented with 95% confidence intervals and without adjustment for multiple comparisons, consistent with the pilot nature of this trial.

### Sample size justification

Based on the primary feasibility outcome of completion rate (≥80% as evidence to proceed to a definitive trial), a target sample size of 40 participants per group yields a 95% Wilson score confidence interval of approximately (64%, 91%) around the 80% completion threshold. This level of precision is considered acceptable for informing the design of a future phase III trial.The progression criteria to a future definitive trial are: protocol-defined intervention completion rate ≥80% and recruitment rate ≥50% of target per month. A completed SPIRIT 2013 checklist is provided as a supplementary file.

### Data collection and management

All clinical data will be collected by research assistants and recorded in pre-designed electronic case report forms. Written informed consent documents will be stored in a locked cabinet, and only authorized investigators will have access to the study data. All procedures have been established to ensure data protection and confidentiality.

### Dissemination

The study results will be presented at international conferences and submitted to peer-reviewed journals for publication.

### Patient and public involvement

Patients and the public were not involved in the design, conduct, or outcome measures of this study.

## Discussion

Radiotherapy (RT) plays a crucial role in both curative and palliative treatment of esophageal cancer. However, its frequent and severe side effect—radiation-induced lung injury (RILI)—often leads to delays or interruptions in anticancer therapy, thereby compromising local tumor control, reducing quality of life, and even increasing mortality risk. Hence, there is an urgent need to develop effective prophylactic agents to mitigate radiation-related toxicity and improve treatment outcomes.

To date, the only radioprotector approved by the US Food and Drug Administration (FDA) is amifostine; however, its modest efficacy and significant side effects limit its clinical utility. In this context, the development of novel agents for preventing RILI has garnered considerable attention, particularly drugs that may also be applicable in the management of recurrent interstitial pulmonary fibrosis.

Tetrandrine, a bisbenzylisoquinoline alkaloid (molecular formula C38H_42_N_2_O_6_) extracted from the root of Stephania tetrandra, functions as a calcium channel blocker. Studies have shown that it exhibits broad biological activity by modulating multiple signaling pathways involved in oxidative stress, autophagy, multidrug resistance, caspase activation, and cell cycle arrest (8). In cellular and animal models, tetrandrine has been demonstrated to alleviate oxidative stress and downregulate levels of inflammatory cytokines such as TNF-α and IL-6—both of which are closely associated with the development of radiation pneumonitis. TNF-α acts as a key initiator of inflammatory responses, triggering cytokine cascades and promoting localized inflammation in radiation-induced lung injury. IL-6 plays a central role in inflammation and immune regulation, and meta-analyses have indicated its involvement in the pathogenesis of radiation pneumonitis. By inhibiting these factors, tetrandrine may contribute to a reduced incidence of radiation pneumonitis.

Furthermore, tetrandrine has demonstrated antifibrotic potential: *in vitro* experiments confirm its ability to suppress TGF-β1 transcription and signaling. TGF-β1, a critical regulator of cell proliferation, differentiation, and extracellular matrix deposition, is widely recognized as being closely associated with radiation-induced pulmonary fibrosis. Tetrandrine has already shown antifibrotic effects in hepatic and pulmonary interstitial fibrosis models, inhibits fibroblast proliferation and collagen synthesis, and is currently used clinically in the treatment of silicosis-related fibrosis.

Current research on radiation-induced lung injury (RILI) largely originates from studies in lung cancer. However, significant differences exist in anatomical structure, pathogenesis, and treatment strategies between esophageal cancer and lung cancer. According to the National Comprehensive Cancer Network (NCCN) Guidelines and relevant expert consensus, the recommended dose for neoadjuvant radiotherapy in esophageal and esophagogastric junction cancers is DT 41.4–50.4 Gy, while definitive radiotherapy is delivered at DT 50–50.4 Gy, with a lung V20 constraint of ≤ 20%. In contrast, for non-small cell lung cancer (NSCLC), the typical neoadjuvant radiotherapy dose ranges from DT 45–54 Gy, and definitive radiotherapy is administered at DT 60–70 Gy, with a lung V20 limit of 35%–40%.

This marked difference in radiotherapy intensity between esophageal cancer and lung cancer limits the direct application of predictive models for radiation pneumonitis (RP) derived from lung cancer to esophageal cancer patients. Therefore, there is a clear need for more RILI studies specifically focused on esophageal cancer to better guide the prevention and management of RILI in this patient population.

Regarding radiotherapy dosing, the Chinese Society of Clinical Oncology (CSCO) Guidelines for Esophageal Cancer (2024) (12) recommend a neoadjuvant radiation dose of DT 40 –45 Gy, and a definitive concurrent chemoradiation dose of DT 50–60 Gy. For patients being considered for salvage surgery, the dose should be limited to DT 50–50.4 Gy. Although a dose of 60 Gy remains widely used in many Chinese centres for definitive treatment, the guidelines highlight emerging evidence supporting lower doses. The present study adopts 50–50.4 Gy, consistent with international standards, to enable direct comparability with global literature and to minimize the risk of radiation-induced lung injury.

The phase III CROSS trial (13) established that a neoadjuvant dose of 40–41.4 Gy provides significant overall survival benefit, which persists for at least 10 years of follow-up. Furthermore, two major phase III studies—the ARTDECO trial (14) and work by Xu et al. (15)—demonstrate comparable efficacy between 50 Gy and 60 Gy, with the higher dose associated with increased toxicity, such as radiation pneumonitis.Both the NCCN Guidelines (2025) and ESMO Guidelines (2022) recommend a dose of DT 50.4 Gy for definitive CCRT. The ESMO Guidelines further emphasize that no current evidence supports improved local control or overall survival with doses exceeding 50.4 Gy. The divergence in dose recommendations between Chinese and Western guidelines may be attributed to differences in histologic distribution: over 90% of esophageal cancers in China are squamous cell carcinomas (per CSCO 2022 commentary), whereas adenocarcinoma predominates in Western countries (ESMO 2022).

The landmark CROSS trial, which established the standard of care in Western countries, demonstrated that neoadjuvant radiotherapy at DT 41.4–50.4 Gy and definitive radiotherapy at DT 50–50.4 Gy achieved high rates of pathologic complete response (pCR) with manageable toxicity. In contrast, the common use of 60 Gy for esophageal squamous cell carcinoma (ESCC) in China is partly extrapolated from experience in head and neck squamous cell carcinoma, lacking high-level evidence specific to esophageal cancer.

Adopting a dose framework consistent with international standards (DT 50–50.4 Gy) enables direct comparability with high-quality RILI data largely derived from Western studies and will provide high-level clinical evidence to optimize radiotherapy dosing for esophageal cancer in China. Moreover, limiting the dose to 50–50.4 Gy may facilitate safer subsequent salvage surgery when indicated. For these reasons, this study will adopt the internationally accepted dose range: neoadjuvant radiotherapy at DT 41.4–50.4 Gy and definitive radiotherapy at DT 50–50.4 Gy.

For lung tissue dose constraints, this trial will follow the Chinese Guidelines for Radiotherapy of Esophageal Cancer (2024), with limits set at V20≤ 30%, V5 < 63%, and mean lung dose (MLD) < 20Gy (associated with approximately 20% risk of grade≥2 radiation pneumonitis under these constraints). By comparison, the NCCN Guidelines recommend stricter thresholds: V20 ≤ 20%, V5 ≤ 50%, and MLD < 20 Gy.

This discrepancy may be explained by several factors. First, the predominance of mid- to upper-thoracic ESCC in China necessitates larger target volumes encompassing the entire mediastinum and supraclavicular nodal regions, inevitably irradiating more lung tissue. Slightly relaxed constraints help ensure adequate target coverage while minimizing severe RILI risk. In contrast, Western patients more frequently present with distal adenocarcinoma/GEJ tumors, where irradiated lung volume is smaller, enabling stricter dosimetric goals.

Second, differences in standard treatment approaches influence toxicity profiles. In the West, neoadjuvant chemoradiation to 41.4 Gy followed by surgery is common. The lower radiation dose confers a lower absolute risk of RILI, permitting stricter constraints. In China, where definitive CCRT to 60 Gy is frequently used, the risk of pneumonitis is substantially higher. Enforcing V20 ≤ 20% under these conditions could compromise target coverage and tumor control.

Thus, dose constraints in China and the West are derived from distinct clinical populations, differing in radiobiological susceptibility and toxicity expression across dose levels. The constraint of V20 ≤ 30% represents a pragmatic balance between efficacy and safety within the Chinese clinical context.
